# The Cincinnati Dental College Society

**Published:** 1865-11

**Authors:** 


### THE CINCINNATI DENTAL COLLEGE SOCIETY.

This Association composed of graduates and students of the Ohio Dental College, has just been reorganized.

This Society was put in operation by the students of this Institution at its last session. The object is for the discussion and elaboration of those subjects, that are presented during their course of study, and for the transaction of such other business, as may be of interest and profit, This is right. It is gratifying to see those who are about to enter the profession lay hold of the most profitable means of development, and use them with vigor.

We shall hope hereafter to give a more full account of the doings of this society. Its meetings are held weekly and we understand all members of the profession are invited, and especially the junior members. There is at every meeting, some practical subject up for discussion.

We hope the college will never be without its student's society.

